# Independent and joint associations of cardiorespiratory fitness and lower-limb muscle strength with cardiometabolic risk in older adults

**DOI:** 10.1371/journal.pone.0292957

**Published:** 2023-10-23

**Authors:** Marcyo Camara, Kenio C. Lima, Yuri A. Freire, Gabriel C. Souto, Geovani A. D. Macêdo, Raissa de M. Silva, Ludmila L. P. Cabral, Rodrigo A. V. Browne, Telma M. A. M. Lemos, Debra L. Waters, Edgar R. Vieira, Todd M. Manini, Eduardo Caldas Costa

**Affiliations:** 1 ExCE Research Group, Department of Physical Education, Federal University of Rio Grande do Norte, Natal, Rio Grande do Norte, Brazil; 2 Graduate Program in Health Sciences, Federal University of Rio Grande do Norte, Natal, Rio Grande do Norte, Brazil; 3 Department of Dentistry, Federal University of Rio Grande do Norte, Natal, Rio Grande do Norte, Brazil; 4 Department of Clinical and Toxicological Analysis, Federal University of Rio Grande do Norte, Natal, Rio Grande do Norte, Brazil; 5 Department of Medicine and School of Physiotherapy, University of Otago, Dunedin, Otago, New Zealand; 6 Department of General Internal Medicine/Geriatrics, University of New Mexico, Albuquerque, New Mexico, United States of America; 7 Department of Physical Therapy, Florida International University, Miami, Florida, United States of America; 8 Institute on Aging, University of Florida, Gainesville, Florida, United States of America; 9 Department of Health Outcomes and Biomedical Informatics, University of Florida, Gainesville, Florida, United States of America; Saga University, JAPAN

## Abstract

The aim of this study was to investigate the independent and joint associations of low cardiorespiratory fitness and lower-limb muscle strength with cardiometabolic risk in older adults. A total of 360 community-dwelling older adults aged 60–80 years participated in this cross-sectional study. Cardiometabolic risk was based on the diagnosis of Metabolic Syndrome and poor Ideal Cardiovascular Health according to the American Heart Association guidelines. Cardiorespiratory fitness and lower-limb muscle strength were estimated using the six-minute walk and the 30-second chair stand tests, respectively. Participants in the 20^th^ percentile were defined as having low cardiorespiratory fitness and lower-limb muscle strength. Poisson’s regression was used to determine the prevalence ratio (PR) and 95% confidence intervals (CI) of Metabolic Syndrome and poor Ideal Cardiovascular Health. Participants with low cardiorespiratory fitness alone and combined with low lower-limb muscle strength were similarly associated with a higher risk for Metabolic Syndrome (PR 1.27, 95% CI 1.09–1.48, and PR 1.32, 95% CI 1.10–1.58, respectively), and poor Ideal Cardiovascular Health (PR 1.76, 95% CI 1.25–2.47, and PR 1.65, 95% CI 1.19–2.28, respectively). Low lower-limb muscle strength alone was not associated with a higher risk for either Metabolic Syndrome or poor Ideal Cardiovascular Health (PR 1.23, 95% CI 0.81–1.87, and PR 1.11, 95% CI 0.89–1.37, respectively). Low cardiorespiratory fitness alone or combined with low lower-limb muscle strength, but not low lower-limb muscle strength alone, was associated with a higher cardiometabolic risk in older adults. The assessment of physical fitness may be a “window of opportunity” to identify youngest-old adults with a high cardiovascular disease risk.

## Introduction

Cardiovascular diseases (CVD) are the main cause of mortality in older adults [1, 2]. Understanding the risk factors and implementing countermeasures are pivotal to avoid adverse CVD events [3]. Metabolic Syndrome (MetS) is a combination of three or more biological risk factors for CVD (i.e., high blood pressure, fasting glucose, total cholesterol, triglycerides, and waist circumference) [4]. The Ideal Cardiovascular Health (ICH) concept proposed by the American Heart Association [5] includes both biological and behavioral components (i.e., adequate blood pressure, glucose, total cholesterol, no smoking, body mass index, physical activity level, and diet habits). Importantly, the ICH metric incorporates well-recognized behavioral risk factors, including body mass index, physical activity, and diet, which are not accounted for in traditional clinical tools used for CVD screening, such as the Framingham risk score [5]. Previous studies have reported that MetS and poor ICH are independent predictors of CVD morbidity and mortality [6–8].

In addition to CVD risk algorithms such as MetS and ICH, physical fitness is a strong predictor of cardiometabolic health [9]. Aging reduces cardiorespiratory fitness and muscle strength [10, 11] and both low cardiorespiratory fitness and muscle strength are independently associated with increased cardiometabolic risk in older adults [12, 13]. Interestingly, some studies have suggested a potential additive effect of combined low cardiorespiratory fitness and muscle strength to predict adverse cardiometabolic outcomes, such as arterial hypertension [14], diabetes [15], and MetS [16]. Of note, data from UK Biobank Study [17] showed an additive effect of combining cardiorespiratory fitness and muscle strength to predict CVD mortality compared to cardiorespiratory fitness or muscle strength alone in middle-aged and older adults. However, these findings do not extend to CVD risk alone; in other words, it was not possible to identify individuals’ risk of developing CVD based on specific screening tools. Investigations on the independent and combined associations of cardiorespiratory fitness and muscle strength with cardiometabolic risk in older adults may contribute to identify fitness-related phenotypes associated with a higher CVD risk. This is important because incorporating fitness levels into established CVD screening algorithms enhances the accuracy of risk prediction for both CVD and mortality [18]. Such investigations also provide insights about which type of physical exercise (aerobic, resistance, or combined) may optimize the reduction of CVD risk in this population. Therefore, this study aimed to investigate the independent and joint associations of low cardiorespiratory fitness and low muscle strength with cardiometabolic risk, using CVD risk algorithms endorsed by the American Heart Association (MetS and ICH) [4], in older adults. It was hypothesized that older adults with combined low cardiorespiratory fitness and muscle strength would have the highest cardiometabolic risk.

## Methods

This cross-sectional study was reported in accordance with the STROBE (STrengthening the Reporting of OBServational Studies in Epidemiology) statement guidelines [19]. The study was conducted at the Onofre Lopes University Hospital (HUOL) and at the Department of Physical Education of the Federal University of Rio Grande do Norte (UFRN) between October 2018 and April 2019. This study was approved by the Ethics Committee in Research of HUOL (Protocol Number: 2.603.422/2018).

### Participants

**Fig 1:**
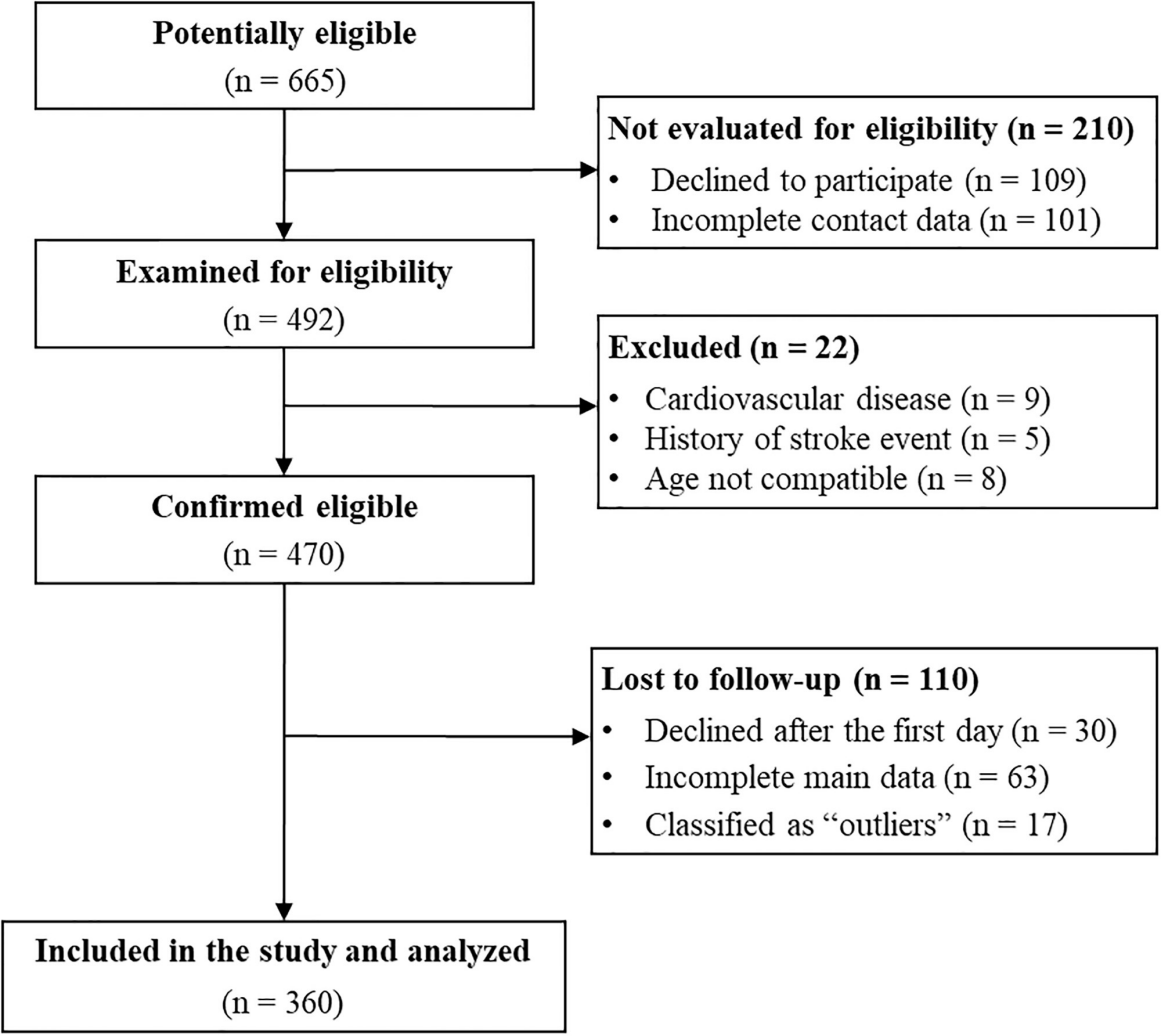
Study flowchart.

Community-dwelling adults aged 60–80 years from the city of Natal, RN, Brazil were recruited by advertisements on radio, e-flyers in social medias (WhatsApp, Instagram, and Facebook), healthcare units, and community centers for older adults. The informed consent was provided in written form and was read and signed by all participants upon their arrival for data collection. All participants received an identification code at the onset of recruitment. Only the research staff directly engaged in data collection had access to participants’ names. Inclusion criteria were: i) no history of CVD or major adverse cardiovascular events (i.e., acute myocardial infarction, stroke, coronary artery disease, arrhythmias, or peripheral vascular disease); ii) no musculoskeletal limitations to perform exercise; iii) no acute diabetes- or hypertension-related decompensations (i.e., fasting glucose ≥ 300 mg/dL; blood pressure ≥ 160/105 mmHg). The sample size was determined from a preliminary study [20] about the prevalence of MetS in older adults with and without low cardiorespiratory fitness and muscle strength (prevalence ratio for MetS of 1.45 in older adults with combined low cardiorespiratory fitness and muscle strength compared to those with normal values). Based on these rates, the required sample size was ≥ 324 participants with an alpha error of 5% and power of 80% (G*Power software, version 3.1.9.2). Fig 1 shows the study flowchart.

### Cardiorespiratory fitness and lower-limb muscle strength

Cardiorespiratory fitness was assessed using the six-minute walk test [21]. The participants were instructed to walk the maximum possible distance in a 30-meter course over 6 minutes. The total distance walked was recorded. Previous studies have found a moderate-high correlation between the six-minute walk test and treadmill performance (r = 0.78) and peak oxygen uptake (r = 0.76) [22, 23]. The six-minute walk test has high reproducibility (r = 0.88–0.94) in older adults [22], and it is highly recommended for clinical purposes, considering its simplicity and low cost [18]. Lower-limb muscle strength was assessed using the 30-second chair stand test [21]. In this test, participants sit down and stand up from a chair as many times as possible within 30 seconds. The number of repetitions is recorded. The 30-second chair stand test exhibits moderate-to-high correlation (r = 0.77) with the one-repetition maximum leg press test and demonstrates high test-retest reliability (r = 0.89) [24]. Moreover, the chair stand test correlates with various markers of lower extremity muscle function, including muscle power, gait speed, and agility/dynamic balance [25]. Results below the 20^th^ percentile on the six-minute walk test and 30-second chair stand test from our cohort, based on sex and age (60–69 and 70–80 years), were used to define low cardiorespiratory fitness and low lower-limb muscle strength, respectively. Results equal to or above the 20^th^ percentile on the six-minute walk test and 30-second chair stand test were used to define normal cardiorespiratory fitness and normal muscle strength, respectively.

### Cardiometabolic risk

To assess the cardiometabolic risk, two approaches were used based on the recommendations of the American Heart Association: diagnosed MetS [4] and poor ICH [24]. MetS includes biological risk factors related to cardiometabolic disease and was defined using the American Heart Association criteria [4]; i.e., presence of at least three of the following: (i) waist circumference > 102 cm in males and > 88 cm in females; (ii) systolic blood pressure ≥ 130 mmHg and/or diastolic blood pressure ≥ 85 mmHg and/or on antihypertensive medication; (iii) HDL-cholesterol < 40 mg/dL in males and < 50 mg/dL in females; (iv) triglycerides ≥ 150 mg/dL and/or on lipid-lowering medication; (v) fasting glucose ≥ 110 mg/dL and/or on diabetes medication.

The ICH metrics include both biological and behavioral protective factors against cardiometabolic diseases and was defined following the recommendations of the American Heart Association [24]: i) blood pressure: < 120/80 mmHg without medication; ii) total cholesterol: < 200 mg/dL without medication; iii) fasting glucose: < 100 mg/dL without medication; iv) body mass index (BMI): < 25 kg/m^2^; v) never smoked or stopped smoking for at least 12 months; vi) appropriate intake of at least four of the following items: 1) fresh vegetables and fruits (≥ 2 times/day); 2) grains and oilseeds (≥ 1 time/day); 3) consumption of fish (≥ 3 days/week); 4) consumption of sugary drinks (≤ 4 days/week); 5) consumption of ultra-processed foods (≤ 4 days/week); vii) physical activity: ≥ 75 minutes/week of vigorous physical activity or ≥ 150 minutes/week of moderate physical activity. Poor ICH was defined as not meeting ≥5 of the above-mentioned criteria [25].

After a 12-hour overnight fasting period, blood samples were obtained by venipuncture to determine total cholesterol, HDL-cholesterol, LDL-cholesterol, triglycerides, and fasting glucose. LDL-cholesterol was determined according to the Friedewald formula: (total cholesterol—[HDL + triglycerides/5]). All biochemical tests were determined using commercial kits (Diagnostic Labtest-SA, São Paulo, Brazil) according to the colorimetric method/enzyme (Labtest, Labmax Plenno, Minas Gerais, Brazil). Body mass index was calculated as weight in kilograms divided by height in meters squared (kg/m^2^). Waist circumference (cm) was measured in the midway between the lateral iliac crest and the lowest rib margin at the end of normal expiration twice and the average value was recorded. Resting blood pressure was measured in the sitting position using an oscillometric device (Omron HEM-780-E, Kyoto, Japan) in triplicate, two minutes apart between each measurement. The average value of the last two measurements was used for analysis [26]. Physical activity level was assessed using questions about daily walking frequency, sports activities and recreational/leisure activities contained in the modified Minnesota Leisure Activity Questionnaire [27]. The activities were first classified according to their metabolic equivalents (METs), based on the compendium of physical activity [28]. The intensity of physical activity was classified according to age as “light, moderate or vigorous” [29]. Diet was assessed by a food frequency questionnaire [30], including questions about fruits, vegetables, grains, oil seeds, fishes, sugary beverages, and ultra-processed foods which adapted a list according to food groups and their level of processing according to the review of the food guide for the Brazilian population [31]. The frequency was based on Brazilian guidelines for the prevention of CVD and major adverse cardiovascular events [32, 33]. Both the physical activity level and diet were assessed during face-to-face interviews with the participants.

### Covariates

Socioeconomic information was collected (sex, age, educational level, marital status, ethnicity and family income). Sedentary time was assessed using the Longitudinal Aging Study Amsterdam–Sedentary Behavior Questionnaire [34]. However, the ‘napping’ question was not considered, given that sedentary activities are only considered in the awake period [35]. More than eight hours per day of sedentary time was considered as ‘high sedentary time’ [36].

### Statistical analysis

Continuous and categorical data were expressed as mean ± standard deviation (SD) and absolute and relative (%) rates, respectively. The calculation of the Mahalanobis Distance was used to identify the outliers regarding the outcome variables (i.e., Mahalanobis probability values ≤ 0.005). Independent-sample t-test, one-way ANOVA, Chi-squared and Fisher’s exact tests were used for bivariate analysis. Pearson’s correlation coefficient was used to test the correlation between cardiorespiratory fitness and muscle strength. In the multiple analysis, Poisson’s regression model with robust variance was used to calculate the prevalence ratio (PR) and its 95% confidence interval (IC) for MetS and poor ICH using low cardiorespiratory fitness or low muscle strength as predictors with normal cardiorespiratory fitness and normal muscle strength as reference groups, respectively. For this independent analysis, the models were unadjusted, adjusted for confounders (Model^a^), and adjusted by confounders plus muscle strength in the model with low cardiorespiratory fitness as a predictor and by cardiorespiratory fitness in the model with low muscle strength as a predictor (Model^b^). For joint associations analysis, low cardiorespiratory fitness (but normal muscle strength), low muscle strength (but normal cardiorespiratory fitness), and combined low cardiorespiratory fitness and muscle strength were the predictors (models) and combined normal cardiorespiratory fitness and muscle strength was the reference group. All multiple regression models were adjusted for the independent variables that were different between the groups with and without MetS and poor vs. ideal ICH in the bivariate analyses at p < 0.20 [37]. The following independent variables were considered as potential confounders for MetS and poor ICH: age, sex, alcohol consumption, ethnicity, income, and sedentary time. Only the significant independent variables with a p < 0.10 were retained in the multiple regression models [37], which were age, sex, and sedentary time. The assumptions of Poisson’s regression were verified, including multicollinearity. The Omnibus test was used to verify the goodness of fit of the models; a well-adjusted model is represented by a p < 0.05. The level of significance was set at p < 0.05. Statistical analysis was performed using the IBM SPSS Statistics 25.0 program (IBM, Chicago, IL, USA).

## Results

Three hundred and sixty older adults were included in the final analysis (Fig 1). Seventeen participants were excluded from the study by the Mahalanobis Distance (S1 Table). Most participants were females (72.8%) and had excess weight (overweight: 19.9%; obesity: 37.9%). Almost half of the participants were Caucasian (41.7%), 24.5% had post-secondary education, and 31.2% were living alone. Few participants were current smokers (3.8%), and approximately one-third were ex-smokers (34.9%). Table 1 shows the characteristics of the participants. S2 Table shows the characteristics of the participants according to the cardiorespiratory fitness and lower-limb muscle strength classification. In addition, S3 and S4 Tables display the distribution of the participants according to the cardiorespiratory fitness and lower-limb muscle strength classification and by sex, respectively.

**Table 1:** Characteristics of the participants (n = 360).

	Total	Males	Females	P
n, %	360 (100)	100 (27.2)	260 (72.8)	
Age, years	66 ± 5	66 ± 4	66 ± 5	0.958
White, n (%)	151 (41.9)	41 (41)	110 (42.3)	0.814
Pardo/Black, n (%)	209 (58.1)	59 (59)	150 (57.7)	0.814
Living alone, n (%)	112 (31.2)	10 (10)	102 (39.2)	<0.001
Post-secondary education, n (%)	91 (25.2)	31(31.3)	60 (23.1)	0.109
Body mass index, kg/m^2^	28 ± 5	27 ± 4	29 ± 5	0.035
Waist circumference, cm	95 ± 12	98 ± 13	94 ± 12	0.016
Ex-smokers/smokers, n (%)	141 (39)	54 (54)	87 (33.5)	<0.001
Systolic blood pressure, mmHg	128 ± 16	131 ± 16	126 ± 16	0.010
Diastolic blood pressure, mmHg	71 ± 9	74 ± 9	70 ± 9	<0.001
Triglycerides, mg/dL	150 ± 67	152 ± 66	149 ± 68	0.703
Total cholesterol, mg/dL	204 ± 42	192 ± 39	208 ± 41	0.001
HDL-cholesterol, mg/dL	46 ± 12	42 ± 11	48 ± 12	<0.001
LDL-cholesterol, mg/dL	130 ± 38	122 ± 36	133 ± 39	0.018
Fasting glucose, mg/dL	108 ± 24	112 ± 26	106 ± 23	0.034
Ideal FAV intake, n (%)	265 (73.6)	75 (75)	190 (73.1)	0.711
Ideal fish intake, n (%)	45 (12.5)	16 (16)	29 (11.2)	0.213
Ideal GAOS intake, n (%)	118 (32.8)	34 (34)	84 (32.3)	0.759
Ideal FAUF intake, n (%)	282 (78.3)	84 (84)	198 (76.2)	0.106
Ideal CASD intake, n (%)	354 (98.3)	99 (99)	255 (98.1)	0.540
Sedentary time, h/day	7.2 ± 3.3	7.5 ± 3.7	7.1 ± 3.1	0.377
MVPA, MET·minutes/wk	682 ± 963	862 ± 1312	613 ± 783	0.027
Six-minute walk test, m	495 ± 80	548 ± 80	475 ± 71	<0.001
30-s chair stand test, rep	13 ± 4	15 ± 4	13 ± 3	<0.001

Data are expressed as mean ± standard deviation or absolute and relative rates. The p-value refers to the differences between males and females. Abbreviations: FAV, fruits, and vegetables; GAOS, grains and oil seeds; FAUF, fries and ultra-processed foods; CASD, candies and sugary drinks; MVPA, moderate-vigorous physical activity.

The cut-off points for defining low cardiorespiratory fitness based on the six-minute walk test were as follows: males aged 60–69 < 492 meters; males aged 70–80 < 398 meters; females aged 60–69 < 431 meters; females aged 70–80 < 378 meters. The cut-off points for defining low lower-limb muscle strength based on the 30-second chair stand test were: males aged 60–69 < 12 repetitions; males aged 70–80 < 11 repetitions; females aged 60–69 < 11 repetitions; females aged 70–80 < 10 repetitions.

The prevalence of MetS, poor ICH and their respective individual components are shown in Table 2. The prevalence of MetS and poor ICH was 72.5% and 40.8%, respectively. The prevalence of MetS and high blood pressure was higher in older adults with low cardiorespiratory fitness and combined low cardiorespiratory fitness and lower-limb muscle strength, but not in those with low lower-limb muscle strength when compared to those with normal cardiorespiratory fitness and lower-limb muscle strength (p < 0.05). However, abdominal obesity had higher prevalence in older adults with low cardiorespiratory fitness, low lower-limb muscle strength, and combined low cardiorespiratory fitness and lower-limb muscle strength compared to those with normal cardiorespiratory fitness and lower-limb muscle strength (p < 0.05). In addition, the prevalence of poor ICH, high blood pressure, and BMI was higher in older adults with low cardiorespiratory fitness, low lower-limb muscle strength, and combined low cardiorespiratory fitness and lower-limb muscle strength compared to those with normal cardiorespiratory fitness and lower-limb muscle strength (p < 0.05). In contrast, physical inactivity was more prevalent in older adults with low lower-limb muscle strength and combined low cardiorespiratory fitness and lower-limb muscle strength compared to those with normal cardiorespiratory fitness and lower-limb muscle strength (p < 0.05).

**Table 2:** Prevalence of Metabolic Syndrome and poor Ideal Cardiovascular Health in the community-dwelling older adults (n = 360).

	Total (n = 360)	Normal CRF and MS (n = 264)	Low CRF (n = 30)	Low MS (n = 30)	Low CRF and MS (n = 36)	p*
**Metabolic Syndrome, n (%)**	261 (72.5)	181 (68.6)	26 (86.7)*	23 (76.7)	31 (86.1)*	0.034
High blood pressure	254 (70.6)	173 (65.5)	26 (86.7)*	24 (80.0)	31 (86.1)*	0.006
Abdominal obesity	212 (58.9)	138 (52.3)	24 (80.0)*	21 (70.0)*	29 (80.6)*	<0.001
Low HDL-cholesterol	252 (70.0)	186 (70.5)	22 (73.3)	20 (66.7)	24 (66.7)	0.911
High triglycerides	216 (60.0)	154 (58.3)	18 (60.0)	17 (56.7)	27 (75.0)	0.282
High fasting glucose	230 (63.9)	168 (63.6)	21 (70.0)	18 (60.0)	23 (63.9)	0.876
**Poor Ideal Cardiovascular Health, n (%)**	147 (40.8)	96 (36.4)	17 (56.7)*	14 (46.7)*	20 (55.6)*	0.030
High blood pressure	297 (82.5)	208 (78.8)	28 (93.3)*	27 (90.0)*	34 (94.4)*	0.021
High body mass index	283 (78.6)	197 (69.6)	28 (93.3)*	24 (80.0)*	34 (94.4)*	0.008
High total cholesterol	253 (70.3)	187 (70.8)	23 (76.7)	19 (63.3)	24 (66.7)	0.672
High fasting glucose	231 (64.2)	169 (73.2)	21 (70.0)	18 (60.0)	23 (63.9)	0.879
Smoking	28 (7.8)	19 (7.2)	3 (10.0)	2 (6.7)	4 (11.1)	0.876
Poor diet	260 (72.2)	186 (70.5)	26 (86.7)	22 (73.3)	26 (72.2)	0.314
Physical inactivity	169 (46.9)	113 (42.8)	13 (43.3)	19 (63.3)*	24 (66.7)*	0.013

Data are expressed as absolute and relative rates. Abbreviations: CRF, cardiorespiratory fitness; MS, lower-limb muscle strength.

*, statistically different from Normal CRF and MS group. Poor Ideal Cardiovascular Health was defined by the presence of five abnormal metrics of the American Heart Association [25].

Table 3 shows the prevalence ratios for MetS and poor ICH in older adults with low cardiorespiratory fitness or low lower-limb muscle strength compared to those with normal cardiorespiratory fitness and lower-limb muscle strength, respectively. In the multiple-adjusted analysis, low cardiorespiratory fitness was associated with higher prevalence ratios for MetS and poor ICH, even when adjusted for lower-limb muscle strength (p < 0.05). Low lower-limb muscle strength was associated with higher prevalence ratios for MetS and poor ICH (unadjusted model and adjusted model by age, sex, and sedentary time; p < 0.05), but when adjusted by cardiorespiratory fitness the association was not significant (p = 0.927 for MetS; p = 0.831 for ICH). The Pearson’s correlation coefficient between cardiorespiratory fitness and lower-limb muscle strength was moderate (r = 0.580; p< 0,001).

**Table 3:** Independent association of cardiorespiratory fitness and lower-limb muscle strength with Metabolic Syndrome and poor Ideal Cardiovascular Health in community-dwelling older adults (n = 360).

	n (%)	Unadjusted model	p*	Adjusted model^a^	p^†^	Adjusted model^b^	p^†^
**Metabolic Syndrome**							
Cardiorespiratory fitness							
Normal	296 (81.8)	1.00 (reference)		1.00 (reference)		1.00 (reference)	
Low	66 (18.2)	1.24 (1.10; 1.40)	0.001	1.27 (1.12; 1.45)	<0.001	1.20 (1.03; 1.41)	0.019
Muscle strength							
Normal	294 (81.7)	1.00 (reference)		1.00 (reference)		1.00 (reference)	
Low	66 (18.3)	1.16 (1.01; 1.33)	0.030	1.16 (1.01; 1.34)	0.035	1.01 (0.86; 1.17)	0.927
**Poor Ideal Cardiovascular Health**							
Cardiorrespiratory fitness							
Normal	296 (81.8)	1.00 (reference)		1.00 (reference)		1.00 (reference)	
Low	66 (18.2)	1.49 (1.15; 1.94)	0.002	1.65 (1.28; 2.13)	<0.001	1.43 (1.06; 1.91)	0.018
Muscle strength							
Normal	294 (81.7)	1.00 (reference)		1.00 (reference)		1.00 (reference)	
Low	66 (18.3)	1.34 (1.02; 1.76)	0.037	1.35 (1.03; 1.78)	0.031	1.03 (0.76; 1.42)	0.831

Data are expressed as prevalence ratio (PR) and 95% confidence interval (CI).

*Unadjusted Poisson Regression.

^†^Multivariate Poisson regression.

^a^Model (adjusted for age, sex, and sedentary time); Adjustment (Omnibus Test): MetS, p = 0,016; poor ICH, p = 0.001.

^b^Model (adjusted for ^a^Model and lower-limb MS in the CRF analysis and CRF in the lower-limb MS analysis); Adjustment (Omnibus Test): MetS, p = 0,015; poor ICH, p < 0.001. Abbreviations: CRF, cardiorespiratory fitness; MS, lower-limb muscle strength.

**Fig 2:**
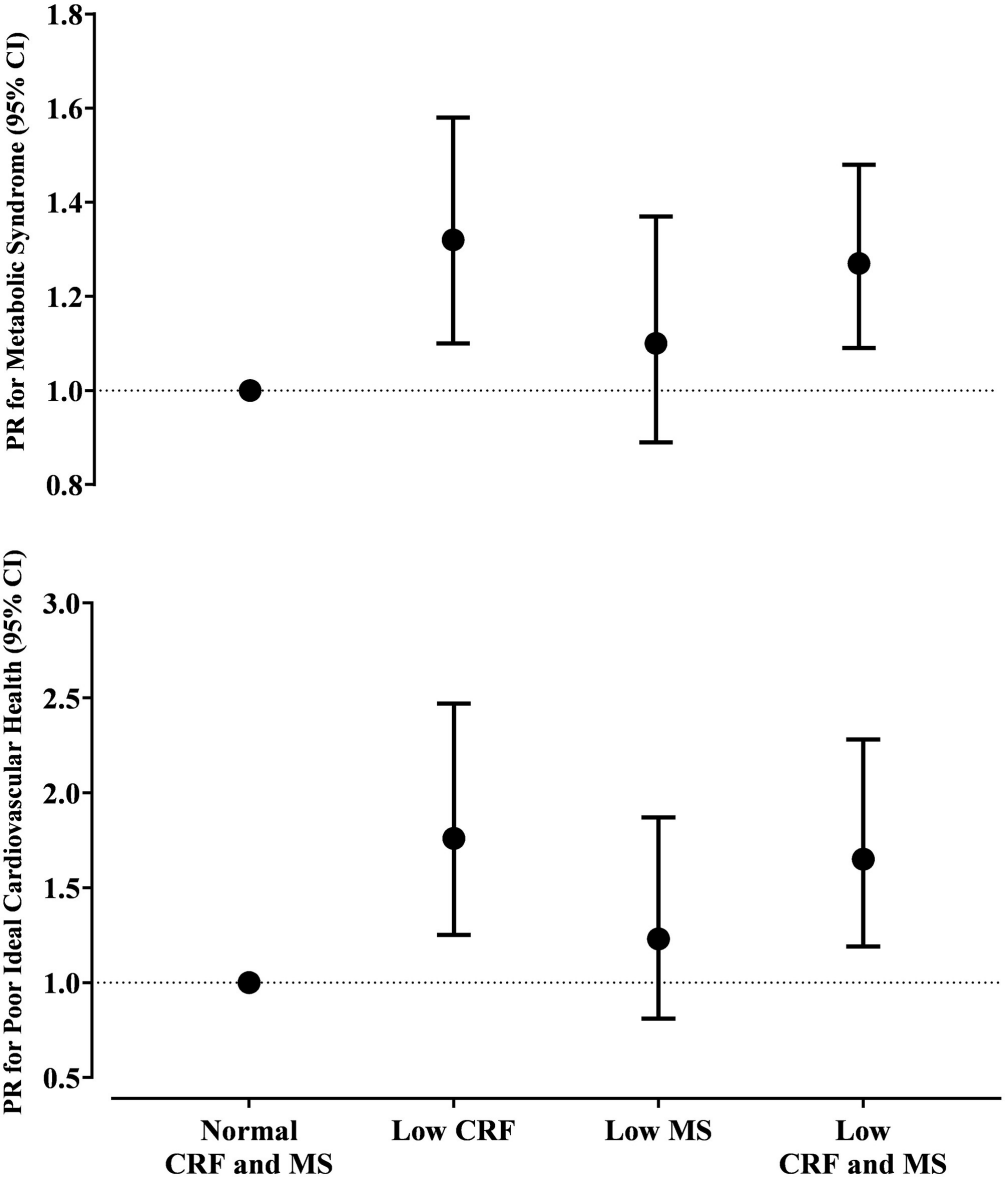
Joint associations of cardiorespiratory fitness and lower-limb muscle strength with Metabolic Syndrome (panel A) and poor Ideal Cardiovascular Health (panel B) in community-dwelling older adults (n = 360). Data are expressed as prevalence ratio (PR) and 95% confidence interval (CI). Models adjusted for age, sex, and sedentary time. Goodness-of-fit (Omnibus test): p < 0,05. Abbreviations: CRF, cardiorespiratory fitness; MS, lower-limb muscle strength.

Fig 2A and S5 Table show the prevalence ratios for MetS in older adults with low cardiorespiratory fitness, low lower-limb muscle strength, and combined low cardiorespiratory fitness and lower-limb muscle strength compared to those with normal cardiorespiratory fitness and lower-limb muscle strength (reference group). In the multiple-adjusted analysis, older adults with low cardiorespiratory fitness (1.32, 95% CI 1.10–1.58) and combined low cardiorespiratory fitness and lower-limb muscle strength (1.27, 95% CI 1.09–1.48) had a higher prevalence ratio for MetS, which was not observed in those with low lower-limb muscle strength.

Fig 2B and S6 Table show the PR for poor ICH in older adults with low cardiorespiratory fitness, low lower-limb muscle strength, and combined low cardiorespiratory fitness and lower-limb muscle strength compared to those with normal cardiorespiratory fitness and lower-limb muscle strength (reference group). In the multiple-adjusted analysis, only older adults with low cardiorespiratory fitness alone (1.76, 95% CI 1.25–2.47) and combined low cardiorespiratory fitness and lower-limb muscle strength (1.65, 95% CI 1.19–2.28) showed a higher PR for poor ICH. No significant associations were observed for those with low lower-limb muscle strength.

## Discussion

Our main finding was that low cardiorespiratory fitness alone or combined with low lower-limb muscle strength, but not low lower-limb muscle strength alone, was associated with a higher cardiometabolic risk in older adults. This result was consistent using both CVD risk algorithms (i.e., MetS and ICH). However, our data suggest that combined low cardiorespiratory fitness and lower-limb muscle strength did not have an additive effect to predict the cardiometabolic risk compared to low cardiorespiratory fitness alone.

Our results suggest that low cardiorespiratory fitness is the driver of the association between poor physical fitness with increased cardiometabolic risk (Fig 2 and Table 3). Data from Aerobics Center Longitudinal Study [38] including mostly middle-aged adults have demonstrated that higher cardiorespiratory fitness was cross-sectionally associated with better cardiovascular health as assessed by ICH. Furthermore, increments of cardiorespiratory fitness were associated with improvements of ICH score [38]. Hassinen et al. [39] reported that low cardiorespiratory fitness, measured by respiratory gas analysis, was associated with higher MetS risk in middle-aged and older adults. Using factor analysis, the authors suggested that low cardiorespiratory fitness was a feature of MetS. Our study adds to the body of evidence showing that the association of low cardiorespiratory fitness with higher cardiometabolic risk in older adults seems to occur independently of the lower-limb muscle strength levels.

The older adults with low cardiorespiratory fitness, alone or combined with low lower-limb muscle strength, had a higher prevalence of high blood pressure, abdominal obesity, high BMI, MetS, and poor ICH compared to those with normal cardiorespiratory fitness and lower-limb muscle strength (Table 2). Cardiorespiratory fitness results from a complex interaction of various systems, mainly cardiovascular, respiratory, and neuromuscular. It is expected that individuals with low cardiorespiratory fitness have an impaired ability to properly integrate the chain involving the above-mentioned systems to translate into physical effort [18]. This may be partially explained by the deleterious effects elicited by the cardiometabolic risk factors, such as high blood pressure and excess body fat. It should be noted that the older adults included in our study were free of CVD. However, based on both CVD risk algorithms used in the current study, our findings suggest that those older adults with low cardiorespiratory fitness may be at a higher risk to develop CVD, which is supported by the findings of the UK Biobank Study [40], i.e., low cardiorespiratory fitness was associated with a higher incidence of CVD over a 6-year follow-up in middle-aged and older adults (aged 40–69 years).

The absence of significant associations of low lower-limb muscle strength with either MetS or poor ICH was unexpected. Cumulative evidence has shown significant associations of low muscle strength (grip strength) with higher cardiometabolic risk ([41], incidence of CVD [42–45], and cardiovascular mortality [45, 46]. Kawamoto et al. [41] and Lee et al. [47] have found that middle-aged and older adults with higher grip strength showed lower risk for MetS. Ramírez-Vélez et al. [48] reported that higher grip strength was associated with better ICH scores in older adults. We believe that some aspects could partially explain the disagreement between ours and previous findings. Although grip strength and chair stand tests are valid and reliable measures of muscle strength [9, 21, 49] there is poor agreement using grip strength and chair stand tests to identify older adults with low muscle strength [50, 51]. These differences between the assessment methods and their implications for muscle strength may also account for some of the discrepancies between the results from our study and previous investigations. More importantly, given that cardiorespiratory fitness was not assessed in the above-mentioned studies [42, 44–47] it is possible that individuals with combined low cardiorespiratory fitness and muscle strength have been included in the “low muscle strength” group. Previous investigations have showed that the adjustment by cardiorespiratory fitness attenuate or even eliminate the association of muscle strength with MetS [52–54], which supports our assumption.

In our study, the presence of low lower-limb muscle strength did not contribute to the increase in cardiometabolic risk of those older adults who already had low cardiorespiratory fitness. Contrary to our findings, Jurca et al. [16] and Kim et al. [54] have reported that combined high cardiorespiratory fitness and muscle strength showed the lowest MetS risk compared to low cardiorespiratory fitness and muscle strength in adult males. In a longitudinal study, Jurca et al. [53] observed that adult males with combined high cardiorespiratory fitness and muscle strength demonstrated the lowest incidence of MetS. More recently, Kim et al. [17] demonstrated that combined high cardiorespiratory fitness and muscle strength showed the lowest risk for CVD in middle-aged and older adults. However, Jurca et al. [16] and Kim et al. [54] have showed that the combination of low cardiorespiratory fitness and high muscle strength was not associated with lower MetS risk, while high cardiorespiratory fitness and low muscle strength was associated. Jurca et al. [53] have showed that the inverse association of muscle strength and MetS was attenuated after adjusting for cardiorespiratory fitness and Kim et al. [17] have found that while cardiorespiratory fitness was inversely associated with CVD mortality when adjusted by muscle strength, the opposite did not occur. Taken together, independent of an additive effect, the combined assessment of cardiorespiratory fitness and muscle strength predicts consistently the CVD risk in older adults, although muscle strength has a lower predictive role.

Noteworthy, most of our participants were youngest-old adults in their first decade of transition between middle to old age, a period in each several aging-related physiological changes that increase the risk for CVD are emerging, particularly in females [55–57]. Indeed, the prevalence of biological and behavioral risk factors for CVD increases dramatically from middle to old age. In Brazil, the prevalence of hypertension (aged 45–64 years: 30.9–49.4%; aged 65+ years: 61%), type 2 diabetes (aged 45–64 years: 11.1–17.1%; aged 65+ years: 28.4%), and physical inactivity (aged 45–64 years: 46.3%-56.5; aged 65+ years: 73%) is > 20% higher in older adults compared to middle-aged individuals [58]. Thus, preventive actions against CVD, including physical exercise, should be strongly encouraged for youngest-old adults identified with low cardiorespiratory fitness alone or combined with low lower-limb muscle strength. To reduce the cardiometabolic risk, our findings support the idea that the exercise programs should be primarily focused in improving the cardiorespiratory fitness. However, as combined exercise training elicits more comprehensive benefits on physical fitness and cardiometabolic health [59–62], both aerobic and resistance exercise training should be considered for older adults identified as high risk for adverse CVD events, i.e., those with low cardiorespiratory fitness alone or in combination with low lower limb muscle strength. This is in accordance with the World Health Organization 2020 guidelines on physical activity and sedentary behavior, which strongly recommend the inclusion of multicomponent exercise programs for older adults [63].

Our study has some strengths and limitations. As strengths, we have used two CVD risk algorithms endorsed by the American Heart Association [4]. Both MetS and ICH score were determined by using direct measures of blood pressure, waist circumference, and blood sample. We have included older adults free of CVD, which allowed us to analyze the association of cardiorespiratory fitness and muscle strength with cardiometabolic risk without the well-known deleterious impact of CVD on these fitness components. As limitations, this is cross-sectional study, which precludes to establish a cause-effect relationship of cardiorespiratory fitness and muscle strength with cardiometabolic risk. Although we have used validated tests to assess cardiorespiratory fitness and muscle strength [9, 18, 22, 23, 49], they are not considered “gold-standard”. In addition, 30-s chair stand test is a specific proxy of lower-limb muscle strength. Physical activity and sedentary time were assessed by self-reported measures, and not by objectively-measured methods, such as accelerometry. Approximately 75% of our sample were females, which precluded an exploration into association analyses by sex. Finally, as one of the inclusion criteria was “without limitations to perform exercise”, we did not include older adults who had very low cardiorespiratory fitness and muscle strength levels. Therefore, our findings should be interpreted with caution and not generalized to other populations.

## Conclusion

Low cardiorespiratory fitness alone or combined with low lower-limb muscle strength, but not low lower-limb muscle strength alone, was associated with a higher cardiometabolic risk in older adults aged 60–80 years old. The assessment of physical fitness may be a “window of opportunity” to identify youngest-old adults with a high CVD risk and to establish preventive countermeasures (i.e., exercise training).

## Supporting information

S1 Table Characteristics of the outliers (n = 17). (DOCX)

S2 Table Characteristics of the participants according to the cardiorespiratory fitness and lower-limb muscle strength classification (n = 360). (DOCX)

S3 Table Distribution of the participants according to the cardiorespiratory fitness and lower-limb muscle strength classification (n = 360). (DOCX)

S4 Table Distribution of the participants according to the cardiorespiratory fitness and lower-limb muscle strength classification by sex (n = 360). (DOCX)

S5 Table Joint associations of cardiorespiratory fitness and lower-limb muscle strength with Metabolic Syndrome in community-dwelling older adults (n = 360). (DOCX)

S6 Table Joint associations of cardiorespiratory fitness and lower-limb muscle strength with poor Ideal Cardiovascular Health in community-dwelling older adults (n = 360). (DOCX)

S1 Checklist STROBE statement—Checklist of items that should be included in reports of observational studies. (DOCX)

## References

[1] Carnethon MR, Biggs ML, Barzilay J, Kuller LH, Mozaffarian D, Mukamal K, et al. Diabetes and coronary heart disease as risk factors for mortality in older adults. Am J Med. 2010;123: 556–e1. doi: 10.1016/j.amjmed.2009.11.023 20569763 PMC3145803

[2] Tsao CW, Aday AW, Almarzooq ZI, Alonso A, Beaton AZ, Bittencourt MS, et al. Heart disease and stroke statistics—2022 update: a report from the American Heart Association. Circulation. 2022;145: e153–e639. doi: 10.1161/CIR.0000000000001052 35078371

[3] Mendis S, Chestnov O. The global burden of cardiovascular diseases: a challenge to improve. Curr Cardiol Rep. 2014;16: 1–9. doi: 10.1007/s11886-014-0486-3 24718672

[4] Grundy SM, Cleeman JI, Daniels SR, Donato KA, Eckel RH, Franklin BA, et al. Diagnosis and management of the metabolic syndrome: an American Heart Association/National Heart, Lung, and Blood Institute scientific statement. Circulation. 2005;112: 2735–2752. doi: 10.1161/CIRCULATIONAHA.105.169404 16157765

[5] Lloyd-Jones D, Hong Y, Labarthe D, Mozaffarian D, Appel L, van Horn L, et al. American Heart Association Strategic Planning Task Force and Statistics Committee. Defining and setting national goats for cardiovascular health promotion and disease reduction: the American Heart Association’s Strategic Impact Goal through 2020 and beyon. Circulation. 2010;121: 586–613. doi: 10.1161/circulationaha.109.192703 20089546

[6] Guo L, Zhang S. Association between ideal cardiovascular health metrics and risk of cardiovascular events or mortality: a meta‐analysis of prospective studies. Clin Cardiol. 2017;40: 1339–1346. doi: 10.1002/clc.22836 29278429 PMC6490399

[7] Hui WS, Liu Z, Ho SC. Metabolic syndrome and all-cause mortality: a meta-analysis of prospective cohort studies. European journal of epidemiology. Springer; 2010. pp. 375–384. doi: 10.1007/s10654-010-9459-z 20425137

[8] Nguyen ATH, Saeed A, Bambs CE, Swanson J, Emechebe N, Mansuri F, et al. Usefulness of the American Heart Association’s ideal cardiovascular health measure to predict long-term major adverse cardiovascular events (from the heart SCORE study). Am J Cardiol. 2021;138: 20–25. doi: 10.1016/j.amjcard.2020.10.019 33065086

[9] Gallo-Villegas JA, Calderón JC. Epidemiological, mechanistic, and practical bases for assessment of cardiorespiratory fitness and muscle status in adults in healthcare settings. Eur J Appl Physiol. 2023; 1–20. doi: 10.1007/s00421-022-05114-y 36683091 PMC10119074

[10] Goodpaster BH, Park SW, Harris TB, Kritchevsky SB, Nevitt M, Schwartz AV, et al. The loss of skeletal muscle strength, mass, and quality in older adults: the health, aging and body composition study. J Gerontol A Biol Sci Med Sci. 2006;61: 1059–1064. doi: 10.1093/gerona/61.10.1059 17077199

[11] Fleg JL, Morrell CH, Bos AG, Brant LJ, Talbot LA, Wright JG, et al. Accelerated longitudinal decline of aerobic capacity in healthy older adults. Circulation. 2005;112: 674–682. doi: 10.1161/CIRCULATIONAHA.105.545459 16043637

[12] Ekblom Ö, Ekblom-Bak E, Rosengren A, Hallsten M, Bergström G, Börjesson M. Cardiorespiratory fitness, sedentary behaviour and physical activity are independently associated with the metabolic syndrome, results from the SCAPIS pilot study. PLoS One. 2015;10: e0131586. doi: 10.1371/journal.pone.0131586 26120842 PMC4486454

[13] Wu H, Liu M, Chi VTQ, Wang J, Zhang Q, Liu L, et al. Handgrip strength is inversely associated with metabolic syndrome and its separate components in middle aged and older adults: a large-scale population-based study. Metabolism. 2019;93: 61–67. Available: doi: 10.1016/j.metabol.2019.01.011 30690038

[14] Crump C, Sundquist J, Winkleby MA, Sundquist K. Interactive effects of physical fitness and body mass index on the risk of hypertension. JAMA Intern Med. 2016;176: 210–216. doi: 10.1001/jamainternmed.2015.7444 26784837 PMC4803286

[15] Crump C, Sundquist J, Winkleby MA, Sieh W, Sundquist K. Physical Fitness Among Swedish Military Conscripts And Long-Term Risk Of Type 2 Diabetes: A Cohort Study. Ann Intern Med. 2016;164: 577–584. doi: 10.7326/m15-2002 26954518 PMC4861045

[16] Jurca R, Lamonte MJ, Church TS, Earnest CP, Fitzgerald SJ, Barlow CE, et al. Associations of muscle strength and aerobic fitness with metabolic síndrome in men. Med Sci Sports Exerc. 2004;36: 1301–1307. 15292736 10.1249/01.mss.0000135780.88930.a9

[17] Kim Y, White T, Wijndaele K, Westgate K, Sharp SJ, Helge JW, et al. The combination of cardiorespiratory fitness and muscle strength, and mortality risk. Eur J Epidemiol. 2018/03/28. 2018;33: 953–964. doi: 10.1007/s10654-018-0384-x 29594847 PMC6153509

[18] Ross R, Blair SN, Arena R, Church TS, Després J-P, Franklin BA, et al. Importance of Assessing Cardiorespiratory Fitness in Clinical Practice: A Case for Fitness as a Clinical Vital Sign: A Scientific Statement From the American Heart Association. Circulation. 2016; CIR-0000000000000461. doi: 10.1161/CIR.0000000000000461 27881567

[19] von Elm E, Altman DG, Egger M, Pocock SJ, Gøtzsche PC, Vandenbroucke JP, et al. The Strengthening the Reporting of Observational Studies in Epidemiology (STROBE) Statement: guidelines for reporting observational studies. International journal of surgery. 2014;12: 1495–1499. doi: 10.1016/j.ijsu.2014.07.013 25046131

[20] Camara M, Browne RAV, Souto GC, Schwade D, Cabral LPL, Macêdo GAD, et al. Independent and combined associations of cardiorespiratory fitness and muscle strength with metabolic syndrome in older adults: A cross-sectional study. Exp Gerontol. 2020;135: 110923. doi: 10.1016/j.exger.2020.110923 32171778

[21] Rikli RE, Jones CJ. Development and validation of a functional fitness test for community-residing older adults. J Aging Phys Act. 1999;7: 129–161. doi: 10.1123/japa.7.2.129

[22] Rikli RE, Jones CJ. The Reliability and Validity of a 6-Minute Walk Test as a Measure of Physical Endurance in Older Adults. J Aging Phys Act. 1998;6: 363–375. doi: 10.1123/japa.6.4.363

[23] Sperandio EF, Arantes RL, Matheus AC, da Silva RP, Lauria VT, Romiti M, et al. Intensity and physiological responses to the 6-minute walk test in middle-aged and older adults: a comparison with cardiopulmonary exercise testing. Brazilian Journal of Medical and Biological Research. 2015;48: 349–353. doi: 10.1590/1414-431X20144235 25714888 PMC4418366

[24] Folsom AR, Yatsuya H, Nettleton JA, Lutsey PL, Cushman M, Rosamond WD, et al. Community prevalence of ideal cardiovascular health, by the American Heart Association definition, and relationship with cardiovascular disease incidence. J Am Coll Cardiol. 2011;57: 1690–1696. doi: 10.1016/j.jacc.2010.11.041 21492767 PMC3093047

[25] Gaye B, Canonico M, Perier M-C, Samieri C, Berr C, Dartigues J-F, et al. Ideal cardiovascular health, mortality, and vascular events in elderly subjects: the three-city study. J Am Coll Cardiol. 2017;69: 3015–3026. doi: 10.1016/j.jacc.2017.05.011 28641790

[26] Barroso WKS, Rodrigues CIS, Bortolotto LA, Mota-Gomes MA, Brandão AA, de M Feitosa AD, et al. Diretrizes Brasileiras de Hipertensão Arterial–2020. Arq Bras Cardiol. 2021;116: 516–658. doi: 10.36660/abc.20201238 33909761 PMC9949730

[27] Lustosa LP, Pereira DS, Dias RC, Britto RR, Parentoni AN, Pereira LSM. Tradução e adaptação transcultural do Minnesota Leisure Time Activities Questionnaire em idosos. Geriatria & Gerontologia. 2011;5: 57–65. https://sbgg.org.br/wp-content/uploads/2014/10/2011-2.pdf#page=9

[28] Ainsworth BE, Haskell WL, Herrmann SD, Meckes N, Bassett DR, Tudor-Locke C, et al. 2011 Compendium of Physical Activities: a second update of codes and MET values. Med Sci Sports Exerc. 2011;43: 1575–1581. doi: 10.1249/MSS.0b013e31821ece12 21681120

[29] Garber CE, Blissmer B, Deschenes MR, Franklin BA, Lamonte MJ, Lee IM, et al. American College of Sports Medicine position stand. Quantity and quality of exercise for developing and maintaining cardiorespiratory, musculoskeletal, and neuromotor fitness in apparently healthy adults: guidance for prescribing exercise. Med Sci Sports Exerc. 2011;43: 1334. doi: 10.1249/mss.0b013e318213fefb 21694556

[30] Ribeiro AB, Cardoso MA. Construção de um questionário de freqüência alimentar como subsídio para programas de prevenção de doenças crônicas não transmissíveis. Revista de Nutrição. 2002;15: 239–245. doi: 10.1590/S1415-52732002000200012

[31] Saúde BrasilM da. Guia alimentar para a população brasileira. Ministério da Saúde; 2014.

[32] Faludi AA, Izar MC de O, Saraiva JFK, Chacra APM, Bianco HT, Afiune A, et al. Atualização da diretriz brasileira de dislipidemias e prevenção da aterosclerose–2017. Arq Bras Cardiol. 2017;109: 1–76. doi: 10.5935/abc.20170121 28813069

[33] Santos RD, Gagliardi ACM, Xavier HT, Magnoni CD, Cassani R, Lottenberg AMP, et al. I Diretriz sobre o consumo de gorduras e saúde cardiovascular. Arq Bras Cardiol. 2013;100: 1–40. doi: 10.1590/S0066-782X2013000900001 23598539

[34] Visser M, Koster A. Development of a questionnaire to assess sedentary time in older persons–a comparative study using accelerometry. BMC Geriatr. 2013;13: 1–8. doi: 10.1186/1471-2318-13-80 23899190 PMC3733654

[35] Tremblay MS, Aubert S, Barnes JD, Saunders TJ, Carson V, Latimer-Cheung AE, et al. Sedentary behavior research network (SBRN)–terminology consensus project process and outcome. International journal of behavioral nutrition and physical activity. 2017;14: 1–17. doi: 10.1186/s12966-017-0525-8 28599680 PMC5466781

[36] Ku P-W, Steptoe A, Liao Y, Hsueh M-C, Chen L-J. A cut-off of daily sedentary time and all-cause mortality in adults: a meta-regression analysis involving more than 1 million participants. BMC Med. 2018;16: 1–9. doi: 10.1186/s12916-018-1062-2 29793552 PMC5998593

[37] Hosmer DW, Lemeshow S. Applied Logistic Regression 2nd edn John Wiley & Sons. Inc: New York, NY, USA. 2000; 160–164.

[38] Ross LM, Barber JL, McLain AC, Weaver RG, Sui X, Blair SN, et al. The association of cardiorespiratory fitness and ideal cardiovascular health in the aerobics center longitudinal study. J Phys Act Health. 2019;16: 968–975. doi: 10.1123/jpah.2018-0220 31553947 PMC12486770

[39] Hassinen M, Lakka TA, Savonen K, Litmanen H, Kiviaho L, Laaksonen DE, et al. Cardiorespiratory fitness as a feature of metabolic syndrome in older men and women: the Dose-Responses to Exercise Training study (DR’s EXTRA). Diabetes Care. 2008;31: 1242–1247. doi: 10.2337/dc07-2298 18332159

[40] Tikkanen E, Gustafsson S, Ingelsson E. Associations of fitness, physical activity, strength, and genetic risk with cardiovascular disease: longitudinal analyses in the UK Biobank Study. Circulation. 2018;137: 2583–2591. doi: 10.1161/CIRCULATIONAHA.117.032432 29632216 PMC5997501

[41] Kawamoto R, Ninomiya D, Kasai Y, Kusunoki T, Ohtsuka N, Kumagi T, et al. Handgrip strength is associated with metabolic syndrome among middle-aged and elderly community-dwelling persons. Clin Exp Hypertens. 2016;38: 245–251. doi: 10.3109/10641963.2015.1081232 26818203

[42] Hu X, Mok Y, Ding N, Sullivan KJ, Lutsey PL, Schrack JA, et al. Physical Function and Subsequent Risk of Cardiovascular Events in Older Adults: The Atherosclerosis Risk in Communities Study. J Am Heart Assoc. 2022;11: e025780. doi: 10.1161/JAHA.121.025780 36043511 PMC9496416

[43] Ding N, Ballew SH, Palta P, Schrack JA, Windham BG, Coresh J, et al. Muscle strength and incident cardiovascular outcomes in older adults. J Am Coll Cardiol. 2020;75: 1090–1092. doi: 10.1016/j.jacc.2019.12.050 32138971

[44] Peralta M, Dias CM, Marques A, Henriques-Neto D, Sousa-Uva M. Association between grip strength and the risk of heart diseases among European middle-aged and older adults. Exp Gerontol. 2022; 112014. doi: 10.1016/j.exger.2022.112014 36347359

[45] Celis-Morales CA, Welsh P, Lyall DM, Steell L, Petermann F, Anderson J, et al. Associations of grip strength with cardiovascular, respiratory, and cancer outcomes and all cause mortality: prospective cohort study of half a million UK Biobank participants. bmj. 2018;361. doi: 10.1136/bmj.k1651 29739772 PMC5939721

[46] Leong DP, Teo KK, Rangarajan S, Lopez-Jaramillo P, Avezum A Jr, Orlandini A, et al. Prospective Urban Rural Epidemiology (PURE) Study Investigators. Prognostic value of grip strength: findings from the prospective urban rural epidemiology (PURE) study. Lancet. 2015;386: 266–273. doi: 10.1016/S0140-6736(14)62000-6 25982160

[47] Lee W-J, Peng L-N, Chiou S-T, Chen L-K. Relative handgrip strength is a simple indicator of cardiometabolic risk among middle-aged and older people: a nationwide population-based study in Taiwan. PLoS One. 2016;11: e0160876. doi: 10.1371/journal.pone.0160876 27559733 PMC4999244

[48] Ramírez-Vélez R, Pérez-Sousa MÁ, Cano-Gutierrez CA, Izquierdo M, García-Hermoso A, Correa-Rodríguez M. Association between ideal cardiovascular health score and relative handgrip strength of community-dwelling older adults in Colombia. J Am Med Dir Assoc. 2020;21: 434–436. doi: 10.1016/j.jamda.2019.12.010 31956013

[49] Jones CJ, Rikli RE, Beam WC. A 30-s chair-stand test as a measure of lower body strength in community-residing older adults. Res Q Exerc Sport. 1999;70: 113–119. doi: 10.1080/02701367.1999.10608028 10380242

[50] de Lucena Alves CP, Câmara M, Dantas Macêdo GA, Freire YA, de Melo Silva R, Paulo-Pereira R, et al. Agreement between upper and lower limb measures to identify older adults with low skeletal muscle strength, muscle mass and muscle quality. PLoS One. 2022;17: e0262732. doi: 10.1371/journal.pone.0262732 35061817 PMC8782376

[51] Johansson J, Strand BH, Morseth B, Hopstock LA, Grimsgaard S. Differences in sarcopenia prevalence between upper-body and lower-body based EWGSOP2 muscle strength criteria: The Tromsø study 2015–2016. BMC Geriatr. 2020;20: 1–11. doi: 10.1186/s12877-020-01860-w 33172391 PMC7654146

[52] Wijndaele K, Duvigneaud N, Matton L, Duquet W, Thomis M, Beunen G, et al. Muscular strength, aerobic fitness, and metabolic syndrome risk in Flemish adults. Med Sci Sports Exerc. 2007;39: 233. doi: 10.1249/01.mss.0000247003.32589.a6 17277586

[53] Jurca R, Lamonte MJ, Barlow CE, Kampert JB, Church TS, Blair SN. Association of muscular strength with incidence of metabolic syndrome in men. Med Sci Sports Exerc. 2005;37: 1849. doi: 10.1249/01.mss.0000175865.17614.74 16286852

[54] Kim J, Lee N, Jung SH, Kim E-J, Cho H-C. Independent and joint associations of cardiorespiratory fitness and muscle fitness with metabolic syndrome in Korean men. Metab Syndr Relat Disord. 2011;9: 273–279. doi: 10.1089/met.2010.0138 21438713

[55] Chia CW, Egan JM, Ferrucci L. Age-Related Changes in Glucose Metabolism, Hyperglycemia, and Cardiovascular Risk. Circ Res. 2018;123: 886–904. doi: 10.1161/CIRCRESAHA.118.312806 30355075 PMC6205735

[56] Ryan AS, Nicklas BJ. Age-related changes in fat deposition in mid-thigh muscle in women: relationships with metabolic cardiovascular disease risk factors. Int J Obes. 1999;23: 126–132. doi: 10.1038/sj.ijo.0800777 10078845

[57] Letnes JM, Dalen H, Aspenes ST, Salvesen Ø, Wisløff U, Nes BM. Age-related change in peak oxygen uptake and change of cardiovascular risk factors. The HUNT Study. Prog Cardiovasc Dis. 2020;63: 730–737. doi: 10.1016/j.pcad.2020.09.002 32971113

[58] da Silva LES, Gouvêa E de CDP, Stopa SR, Tierling VL, Sardinha LMV, Macario EM, et al. Data resource profile: Surveillance system of risk and protective factors for chronic diseases by telephone survey for adults in Brazil (Vigitel). Int J Epidemiol. 2021;50: 1058–1063. doi: 10.1093/ije/dyab104 34050649

[59] Waters DL, Aguirre L, Gurney B, Sinacore DR, Fowler K, Gregori G, et al. Effect of aerobic or resistance exercise, or both, on intermuscular and visceral fat and physical and metabolic function in older adults with obesity while dieting. The Journals of Gerontology: Series A. 2022;77: 131–139. doi: 10.1093/gerona/glab111 33839788 PMC8751785

[60] Villareal DT, Aguirre L, Burke Gurney A, Waters D, Sinacore D, Colombo E, et al. Aerobic or Resistance Exercise, or Both, in Dieting Obese Older Adults. Dieting Obese Older Adults N Engl J Med. 376: 1–20. doi: 10.1056/nejmoa1616338 28514618 PMC5552187

[61] Timmons JF, Minnock D, Hone M, Cogan KE, Murphy JC, Egan B. Comparison of time‐matched aerobic, resistance, or concurrent exercise training in older adults. Scand J Med Sci Sports. 2018;28: 2272–2283. doi: 10.1111/sms.13254 29947107

[62] Schroeder EC, Franke WD, Sharp RL, Lee D. Comparative effectiveness of aerobic, resistance, and combined training on cardiovascular disease risk factors: A randomized controlled trial. PLoS One. 2019;14: e0210292. doi: 10.1371/journal.pone.0210292 30615666 PMC6322789

[63] WHO. WHO guidelines on physical activity and sedentary behaviour. Geneva: World Health Organization. 2020.

