# Supplementary material for: Independent and joint associations of cardiorespiratory fitness and lower-limb muscle strength with cardiometabolic risk in older adults
Source: PLoS One. 2023 Oct 23;18(10):e0292957. doi: 10.1371/journal.pone.0292957 (PMC10593220; doi:10.1371/journal.pone.0292957)
Supplement: S1 Table — (DOCX) [file pone.0292957.s001.docx]

**Supplementary Table 1.** Characteristics of the outliers (n = 17)

|  | **Total** | **Males** | **Females** | **P** |
| --- | --- | --- | --- | --- |
| n, % | 17 (100) | 4 (23.5) | 13 (76.5) |  |
| Age, years | 65 ± 3 | 65 ± 4 | 65 ± 3 | 0.823 |
| Post-secondary education, n (%) | 4 (23.5) | 0 (0.0) | 4 (30.8) | 0.205 |
| Body mass index, kg/m^2^ | 30 ± 7 | 29 ± 2 | 31 ± 8 | 0.663 |
| Waist circumference, cm | 101 ± 17 | 107 ± 11 | 99 ± 18 | 0.375 |
| Ex-smokers/smokers, n (%) | 9 (52.9) | 4 (100) | 5 (38.5) | 0.031 |
| Systolic blood pressure, mmHg | 121 ± 16 | 131 ± 16 | 126 ± 16 | 0.869 |
| Diastolic blood pressure, mmHg | 69 ± 9 | 74 ± 9 | 70 ± 9 | 0.192 |
| Triglycerides, mg/dL | 242 ± 208 | 335 ± 291 | 213 ± 181 | 0.321 |
| Total cholesterol, mg/dL | 216 ± 53 | 216 ± 76 | 216 ± 48 | 0.987 |
| HDL-cholesterol, mg/dL | 46 ± 15 | 40 ± 6 | 48 ± 16 | 0.351 |
| LDL-cholesterol, mg/dL | 147 ± 56 | 153 ± 81 | 145 ± 50 | 0.825 |
| Fasting glucose, mg/dL | 192 ± 106 | 112 ± 26 | 106 ± 23 | 0.034 |
| MVPA, MET·minutes/wk | 229 ± 3164 | 3650 ± 3824 | 1874 ± 2981 | 0.342 |
| Sedentary time, h/day | 8.3 ± 3.6 | 11.4 ± 4.6 | 7.3 ± 2.8 | 0.040 |
| Six-minute walk test, m | 493 ± 71 | 584 ± 52 | 465 ± 43 | 0.001 |
| 30-s chair stand test, rep | 12 ± 2 | 14 ± 2 | 12 ± 2 | 0.039 |

Data are expressed as mean ± standard deviation or absolute and relative rates. Abbreviations: HDL, high density lipoproteins; LDL, low density lipoproteins; MVPA, moderate-vigorous physical activity.
