# Supplementary material for: Independent and joint associations of cardiorespiratory fitness and lower-limb muscle strength with cardiometabolic risk in older adults
Source: PLoS One. 2023 Oct 23;18(10):e0292957. doi: 10.1371/journal.pone.0292957 (PMC10593220; doi:10.1371/journal.pone.0292957)
Supplement: S2 Table — (DOCX) [file pone.0292957.s002.docx]

**Supplementary Table 2.** Characteristics of the participants according to the cardiorespiratory fitness and lower-limb muscle strength classification (n = 360)

|  | **Normal CRF and MS** | **Low CRF** | **Low MS** | **Low CRF and MS** | **P** |
| --- | --- | --- | --- | --- | --- |
| n, % | 264 (73.3) | 30 (8.3) | 30 (8.3) | 36 (10.0) |  |
| Age, years | 66 ± 4 | 68 ± 5 | 65 ± 4 | 68 ± 5 | 0.002 |
| Post-secondary education, n (%) | 74 (81.2) | 4 (4.4) | 6 (6.6) | 7 (7.7) | 0.214 |
| Body mass index, kg/m^2^ | 27.6 ± 4.5 | 30.7 ± 4.2 | 29.6 ± 5.2 | 31.2 ± 4.8 | <0.001 |
| Waist circumference, cm | 93 ± 12 | 103 ± 10 | 100 ± 14 | 101 ± 11 | <0.001 |
| Ex-smokers/smokers, n (%) | 99 (37.5) | 9 (30) | 15 (50) | 18 (50) | 0.202 |
| Systolic blood pressure, mmHg | 127 ± 16 | 134 ± 20 | 126 ± 13 | 125 ± 15 | 0.108 |
| Diastolic blood pressure, mmHg | 71 ± 9 | 74 ± 11 | 72 ± 7 | 69 ± 9 | 0.209 |
| Triglycerides, mg/dL | 147 ± 70 | 157 ± 58 | 145 ± 51 | 168 ± 60 | 0.333 |
| Total cholesterol, mg/dL | 205 ± 43 | 202 ± 35 | 194 ± 34 | 201 ± 45 | 0.603 |
| HDL-cholesterol, mg/dL | 47 ± 12 | 43 ± 11 | 47 ± 12 | 47 ± 13 | 0.414 |
| LDL-cholesterol, mg/dL | 131 ± 39 | 133 ± 38 | 122 ± 30 | 125 ± 37 | 0.545 |
| Fasting glucose, mg/dL | 107 ± 22 | 114 ± 31 | 116 ± 38 | 105 ± 14 | 0.075 |
| MVPA, MET·minutes/wk | 766 ± 1008 | 746 ± 1140 | 384 ± 655 | 281 ± 436 | 0.010 |
| Sedentary time, h/day | 7.3 ± 3.3 | 5.9 ± 2.7 | 7.6 ± 3.8 | 7.5 ± 2.7 | 0.116 |
| Six-minute walk test, m | 521 ± 65 | 405 ± 48 | 501 ± 54 | 373 ± 49 | <0.001 |
| 30-s chair stand test, rep | 15 ± 3 | 13 ± 1 | 10 ± 1 | 8 ± 1 | <0.001 |

Data are expressed as mean ± standard deviation or absolute and relative rates. Abbreviations: CRF, cardiorespiratory fitness; HDL, high density lipoproteins; LDL, low density lipoproteins; MS, lower-limb muscle strength; MVPA, moderate-vigorous physical activity.
