# Supplementary material for: Independent and joint associations of cardiorespiratory fitness and lower-limb muscle strength with cardiometabolic risk in older adults
Source: PLoS One. 2023 Oct 23;18(10):e0292957. doi: 10.1371/journal.pone.0292957 (PMC10593220; doi:10.1371/journal.pone.0292957)
Supplement: S3 Table — (DOCX) [file pone.0292957.s003.docx]

**Supplementary Table 3.** Distribution of the participants according to the cardiorespiratory fitness and lower-limb muscle strength classification (n = 360)

|  | **Normal CRF** | **Low CRF** | **Total** |
| --- | --- | --- | --- |
| **Normal MS** | 264 (73.3) | 30 (8.3) | 294 (81.7) |
| **Low MS** | 30 (8.3) | 36 (10) | 66 (18.3) |
| **Total** | 294 (100) | 66 (100) | 360 (100) |

Data are expressed as absolute (n) and relative (%) rates. Abbreviations: CRF, cardiorespiratory fitness; MS, lower-limb muscle strength.
