# Supplementary material for: Independent and joint associations of cardiorespiratory fitness and lower-limb muscle strength with cardiometabolic risk in older adults
Source: PLoS One. 2023 Oct 23;18(10):e0292957. doi: 10.1371/journal.pone.0292957 (PMC10593220; doi:10.1371/journal.pone.0292957)
Supplement: S4 Table — (DOCX) [file pone.0292957.s004.docx]

**Supplementary Table 4.** Distribution of the participants according to the cardiorespiratory fitness and lower-limb muscle strength classification by sex (n = 360)

|  | **Males** | |  | **Females** | |  |
| --- | --- | --- | --- | --- | --- | --- |
|  | **Normal CRF** | **Low CRF** | **Total** | **Normal CRF** | **Low CRF** | **Total** |
| **Normal MS** | 70 (87.5) | 12 (60) | 82 (82) | 194 (90.6) | 18 (39.1) | 212 (81.5) |
| **Low MS** | 10 (12.5) | 8 (40) | 18 (18) | 20 (9.4) | 28 (60.9) | 48 (18.5) |
| **Total** | 80 (80) | 20 (20) | 100 (100) | 214 (82.3) | 46 (17.7) | 260 (100) |

Data are expressed as absolute (n) and relative (%) rates. Abbreviations: CRF, cardiorespiratory fitness; MS, lower-limb muscle strength.
