# Supplementary material for: Independent and joint associations of cardiorespiratory fitness and lower-limb muscle strength with cardiometabolic risk in older adults
Source: PLoS One. 2023 Oct 23;18(10):e0292957. doi: 10.1371/journal.pone.0292957 (PMC10593220; doi:10.1371/journal.pone.0292957)
Supplement: S5 Table — (DOCX) [file pone.0292957.s005.docx]

**Supplementary Table 5.** Joint associations of cardiorespiratory fitness and lower-limb muscle strength with Metabolic Syndrome in community-dwelling older adults (n = 360)

|  | **n (%)** | **Unadjusted model** | **p*** | **Adjusted model^a^** | **p^†^** |
| --- | --- | --- | --- | --- | --- |
| **Normal CRF and MS** | 264 (73.3) | 1.00 (reference) |  | 1.00 (reference) |  |
| **Low CRF** | 30 (8.3) | 1.26 (1.07; 1.49) | 0.005 | 1.32 (1.10; 1.58) | 0.003 |
| **Low MS** | 30 (8.3) | 1.12 (0.90; 1.38) | 0.305 | 1.11 (0.89; 1.37) | 0.373 |
| **Low CRF and MS** | 36 (10) | 1.26 (1.08; 1.47) | 0.004 | 1.27 (1.09; 1.48) | 0.003 |

Data are expressed as prevalence ratio (PR) and 95% confidence interval (CI). *Unadjusted Poisson Regression. †Multivariate Poisson regression. ^a^Model (adjusted for age, sex, and sedentary time); Adjustment (Omnibus Test): p = 0.047. Abbreviations: CRF, cardiorespiratory fitness; MS, lower-limb muscle strength.
