# Supplementary material for: Independent and joint associations of cardiorespiratory fitness and lower-limb muscle strength with cardiometabolic risk in older adults
Source: PLoS One. 2023 Oct 23;18(10):e0292957. doi: 10.1371/journal.pone.0292957 (PMC10593220; doi:10.1371/journal.pone.0292957)
Supplement: S6 Table — (DOCX) [file pone.0292957.s006.docx]

**Supplementary Table 6.** Joint associations of cardiorespiratory fitness and lower-limb muscle strength with poor Ideal Cardiovascular Health in community-dwelling older adults (n = 360)

|  | **n (%)** | **Unadjusted model** | **p*** | **Adjusted model^a^** | **p^†^** |
| --- | --- | --- | --- | --- | --- |
| **Normal CRF and MS** | 264 (73.3) | 1.00 (reference) |  | 1.00 (reference) |  |
| **Low CRF** | 30 (8.3) | 1.56 (1.10; 2.21) | 0.013 | 1.76 (1.25; 2.47) | 0.001 |
| **Low MS** | 30 (8.3) | 1.28 (0.85;1.94) | 0.237 | 1.23 (0.81; 1.87) | 0.319 |
| **Low CRF and MS** | 36 (10) | 1.53 (1.09; 2.13) | 0.013 | 1.65 (1.19; 2.28) | 0.003 |

Data are expressed as prevalence ratio (PR) and 95% confidence interval (CI). *Unadjusted Poisson Regression. †Multivariate Poisson regression. ^a^Model (adjusted for age, sex, and sedentary time); Adjustment (Omnibus Test): p = 0.003. Abbreviations: CRF, cardiorespiratory fitness; MS, lower-limb muscle strength. Poor Ideal Cardiovascular Health was defined by the presence of five abnormal metrics of the American Heart Association [25].
