# Supplementary material for: Independent and joint associations of cardiorespiratory fitness and lower-limb muscle strength with cardiometabolic risk in older adults
Source: PLoS One. 2023 Oct 23;18(10):e0292957. doi: 10.1371/journal.pone.0292957 (PMC10593220; doi:10.1371/journal.pone.0292957)
Supplement: S1 Checklist — (DOCX) [file pone.0292957.s007.docx]

STROBE Statement—checklist of items that should be included in reports of observational studies

|  | | | Item No. | Recommendation | Page  No. | | | Relevant text from manuscript |
| --- | --- | --- | --- | --- | --- | --- | --- | --- |
| **Title and abstract** | | | 1 | (*a*) Indicate the study’s design with a commonly used term in the title or the abstract | 2 | | | A total of 360 community-dwelling older adults aged 60-80 years participated in this cross-sectional study |
|  |  |  |  | (*b*) Provide in the abstract an informative and balanced summary of what was done and what was found | 2 | | | Low cardiorespiratory fitness alone or combined with low muscle strength, but not low muscle strength alone, was associated with a higher cardiometabolic risk in older adults. |
| Introduction | | | | | | | |  |
| Background/rationale | | | 2 | Explain the scientific background and rationale for the investigation being reported | 3 | | | both low cardiorespiratory fitness and muscle strength are independently associated with increased cardiometabolic risk in older adults |
| Objectives | | | 3 | State specific objectives, including any prespecified hypotheses | 3 | | | It was hypothesized that older adults with combined low cardiorespiratory fitness and muscle strength would have the highest cardiometabolic risk. |
| Methods | | | | | | | |  |
| Study design | | | 4 | Present key elements of study design early in the paper | 3 | | | This cross-sectional study was reported in accordance with the STROBE (STrengthening the Reporting of OBServational Studies in Epidemiology) statement guidelines. |
| Setting | | | 5 | Describe the setting, locations, and relevant dates, including periods of recruitment, exposure, follow-up, and data collection | 3 | | | The study was conducted at the Onofre Lopes University Hospital (HUOL) and at the Department of Physical Education of the Federal University of Rio Grande do Norte (UFRN) between October 2018 and April 2019. This study was approved by the Ethics Committee in Research of HUOL (Protocol Number: 2.603.422/2018). The design and statistical analyzes of this study were conducted between the years 2021 and 2022. |
| Participants | | | 6 | (*a*) *Cohort study*—Give the eligibility criteria, and the sources and methods of selection of participants. Describe methods of follow-up  *Case-control study*—Give the eligibility criteria, and the sources and methods of case ascertainment and control selection. Give the rationale for the choice of cases and controls  *Cross-sectional study*—Give the eligibility criteria, and the sources and methods of selection of participants | 4 | | | Community-dwelling adults aged 60-80 years from the city of Natal, RN, Brazil were recruited by advertisements on radio, e-flyers in social medias (WhatsApp, Instagram, and Facebook), healthcare units, and community centers for older adults. Inclusion criteria were: i) no history of CVD or major adverse cardiovascular events (i.e., acute myocardial infarction, stroke, coronary artery disease, arrhythmias, or peripheral vascular disease); ii) no musculoskeletal limitations to perform exercise; iii) no acute diabetes or hypertension-related decompensations (i.e., fasting glucose ≥ 300 mg/dL; blood pressure ≥ 160/105 mmHg). |
|  |  |  |  | (*b*) *Cohort study*—For matched studies, give matching criteria and number of exposed and unexposed  *Case-control study*—For matched studies, give matching criteria and the number of controls per case |  | | |  |
| Variables | | | 7 | Clearly define all outcomes, exposures, predictors, potential confounders, and effect modifiers. Give diagnostic criteria, if applicable | 4 | | | Socioeconomic information was collected (sex, age, educational level, marital status, ethnicity and family income). |
| Data sources/ measurement | | | 8* | For each variable of interest, give sources of data and details of methods of assessment (measurement). Describe comparability of assessment methods if there is more than one group | 4 | | | To assess the cardiometabolic risk, two approaches were used based on the recommendations of the American Heart Association: diagnosed MetS (4) and poor ICH (26). |
| Bias | | | 9 | Describe any efforts to address potential sources of bias | 4 and 5 | | |  |
| Study size | | | 10 | Explain how the study size was arrived at | 4 | | | The sample size was determined from a preliminary study (20) about the prevalence of MetS of older adults with and without low cardiorespiratory fitness and low muscle strength (prevalence ratio for MetS of 1.45 in older adults with combined low cardiorespiratory fitness and muscle strength compared to those with normal values). Based on these rates, the required sample size was ≥ 324 participants with an alpha error of 5% and power of 80% (G*Power software, version 3.1.9.2). |
| Quantitative variables | | 11 | | Explain how quantitative variables were handled in the analyses. If applicable, describe which groupings were chosen and why | 6 | |  | |
| Statistical methods | | 12 | | (*a*) Describe all statistical methods, including those used to control for confounding | 6 | |  | |
|  |  |  |  | (*b*) Describe any methods used to examine subgroups and interactions | 6 | |  | |
|  |  |  |  | (*c*) Explain how missing data were addressed | 6 | |  | |
|  |  |  |  | (*d*) *Cohort study*—If applicable, explain how loss to follow-up was addressed  *Case-control study*—If applicable, explain how matching of cases and controls was addressed  *Cross-sectional study*—If applicable, describe analytical methods taking account of sampling strategy | 6 | |  | |
|  |  |  |  | (*e*) Describe any sensitivity analyses | 6 | |  | |
| Results | | | | | | | | |
| Participants | | 13* | | (a) Report numbers of individuals at each stage of study—eg numbers potentially eligible, examined for eligibility, confirmed eligible, included in the study, completing follow-up, and analysed | 6 | |  | |
|  |  |  |  | (b) Give reasons for non-participation at each stage | 6 | |  | |
|  |  |  |  | © Consider use of a flow diagram | 6 and 22 | | This is the figure 1. | |
| Descriptive data | | 14* | | (a) Give characteristics of study participants (eg demographic, clinical, social) and information on exposures and potential confounders | 8 | | It can be checked in the Table 1. | |
|  |  |  |  | (b) Indicate number of participants with missing data for each variable of interest | 22 | | It can be checked in the figure 1. | |
|  |  |  |  | (c) *Cohort study*—Summarise follow-up time (eg, average and total amount) |  | |  | |
| Outcome data | | 15* | | *Cohort study*—Report numbers of outcome events or summary measures over time |  | |  | |
|  |  |  |  | *Case-control study—*Report numbers in each exposure category, or summary measures of exposure |  | |  | |
|  |  |  |  | *Cross-sectional study—*Report numbers of outcome events or summary measures | 7 | | The prevalence of MetS, poor ICH and their respective individual components are shown in Table 2. The prevalence of MetS and poor ICH was 72.5% and 40.8%, respectively. | |
| Main results | | 16 | | (*a*) Give unadjusted estimates and, if applicable, confounder-adjusted estimates and their precision (eg, 95% confidence interval). Make clear which confounders were adjusted for and why they were included | 10 | | It can be checked in the table 3. | |
|  |  |  |  | (*b*) Report category boundaries when continuous variables were categorized | 8 | | It can be checked in the table 1. | |
|  |  |  |  | (*c*) If relevant, consider translating estimates of relative risk into absolute risk for a meaningful time period | 10 | |  | |
| Other analyses | 17 | | Report other analyses done—eg analyses of subgroups and interactions, and sensitivity analyses | | 23 | It can be checked in the supplementary tables. | | |
| Discussion | | | | | | | | |
| Key results | 18 | | Summarise key results with reference to study objectives | | 12 |  | | |
| Limitations | 19 | | Discuss limitations of the study, taking into account sources of potential bias or imprecision. Discuss both direction and magnitude of any potential bias | | 14 | Our study has some strengths and limitations. | | |
| Interpretation | 20 | | Give a cautious overall interpretation of results considering objectives, limitations, multiplicity of analyses, results from similar studies, and other relevant evidence | | 12 |  | | |
| Generalisability | 21 | | Discuss the generalisability (external validity) of the study results | | 14 |  | | |
| Other information | | |  | | | | | |
| Funding | 22 | | Give the source of funding and the role of the funders for the present study and, if applicable, for the original study on which the present article is based | | 14 |  | | |

*Give information separately for cases and controls in case-control studies and, if applicable, for exposed and unexposed groups in cohort and cross-sectional studies.

**Note:** An Explanation and Elaboration article discusses each checklist item and gives methodological background and published examples of transparent reporting. The STROBE checklist is best used in conjunction with this article (freely available on the Web sites of PLoS Medicine at http://www.plosmedicine.org/, Annals of Internal Medicine at http://www.annals.org/, and Epidemiology at http://www.epidem.com/). Information on the STROBE Initiative is available at www.strobe-statement.org.
